# Myocardial Restoration: Is It the Cell or the Architecture or Both?

**DOI:** 10.1155/2012/240497

**Published:** 2012-02-16

**Authors:** Duc Thang Vu, Theo Kofidis

**Affiliations:** ^1^Department of Surgery, Yong Loo Lin School of Medicine, National University of Singapore, Singapore 119228; ^2^National University Health System, Department of Cardiac, Thoracic and Vascular Surgery, 1E Lower Kent Ridge Road, NUHS Tower Block, Level 8, Singapore 119228

## Abstract

Myocardial infarction is the leading cause of death in developed countries. Cardiac cell therapy has been introduced to clinical trials for more than ten years but its results are still controversial. Tissue engineering has addressed some limitations of cell therapy and appears to be a promising solution for cardiac regeneration. In this review, we would like to summarize the current understanding about the therapeutic effect of cell therapy and tissue engineering under purview of functional and structural aspects, highlighting actual roles of each therapy towards clinical application.

## 1. Introduction

Ischemic heart disease is the principal cause of chronic heart failure in developed countries. In the USA alone, it causes 400,000 deaths annually [1].

The currently available therapies (i.e., pharmacological, interventional, and surgical methods) are unable to revitalize dead myocardium. Therefore, they cannot halt or reverse the development of congestive heart failure (CHF). Though cardiomyocytes in nonmammalian vertebrate species, like zebrafish, can restore the injured myocardium through proliferation and differentiation, this mechanism is not significant in humans [2]. Cardiac transplantation, the sole definitive therapy with long-term effect for end-stage HF so far, remains limited due to the scarcity of heart donors [3].

Myocardial restoration therapies, including cardiac cell therapy and cardiac tissue engineering, sound promising for a failing heart [4] as their ultimate goals are to regenerate the injured myocardium by robust and viable cells or artificial tissues.

Although 10 years passed since Menasche et al. launched the first clinical trial [5], cardiac cell therapy has not become a well-established medical treatment for postmyocardial infarction (MI) patients. Delivery of cell suspensions to the myocardium is limited by various factors, such as insufficient cell retention and survival [6]. The introduction of cell-cell mechanical interaction systems, in the form of either cell sheets or biomaterial scaffolds [7] has addressed the issues related to poor cell retention and survival. Moreover, this strategy may offer a three-dimensional homogeneous cell delivery plus structural support (scaffold) to the myocardial area of ischemic injury [7]. Yet, there are no clinical studies of this approach.

Though both cardiac cell therapy and tissue engineering have resulted in some improvement of function and structure of the injured heart, it would still be a laborious mission to reproduce the “real” myocardium. In this review, we would like to summarize the latest achievements of regenerative medicine in cardiac repair and obstacles towards a robust cardiac regeneration, under purview of the cardiac structure and the postinfarction cardiac remodelling. We performed a Pubmed search with the keywords “cardiac remodelling,” “myocardial structure,” “cardiac cell therapy,” “cardiac tissue engineering,” “myocardial restoration,” and “surgical ventricular restoration.” Relevant references from all articles reviewed up to June 2011 have been selected for further discussion.

## 2. The Heart Structure and Post-MI Remodelling (Figure 1)

The challenging features of the myocardial restoration are the reproduction of the highly angiotropic and anisotropic three-dimensional muscular structure which has contractile function and mechanics. The optimal regeneration approach needs to address all following features of the heart.

*Cardiomyocytes (CMs) are a decisive component of a functional myocardium* and contribute to the function of the heart by their contraction and intercalated anatomical feature. They are highly resistant to fatigue and rich in myoglobin and mitochondria, featuring a high metabolic demand.

**Figure 1:**
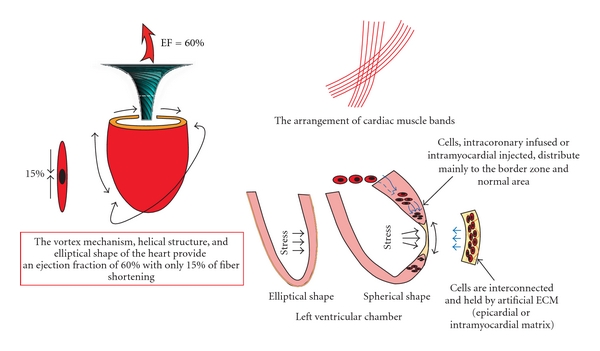
Contraction mechanism of the heart and ventricular wall remodelling after MI. EF: ejection fraction; ECM: extracellular matrix.

The ventricular wall is structured in muscular bands. Its highly asymmetrical and anisotropic architecture facilitates 35–40% increase of thickness of the left ventricular wall in systole, with only 8% thickening of single myofibers [8]. The helical structure is crucial for the production of cardiac work. The contraction of the muscle cells results in multidirectional movement of the ventricular wall, including longitudinal shortening, spiral movement, and thickening of muscle bands, which synergistically produce vortex forces and sheer stress to eject blood. As a result, the spiral assembly can create a left ventricular ejection fraction (LVEF) of 60% with only 15% fibre shortening (Figure 1), while a spherical assembly would only be capable of an LVEF of 30% [9].

*The vascular system of the heart*  is well developed to meet the intense aerobic metabolism of CMs. Blood from the main coronary arteries flows through the penetrating arteries of 20–200 *μ*m at 1 mL per gram of tissue to high dense network of capillary, almost one capillary per cell, with greater distribution in the subendocardial than subepicardial layer. Moreover, the presence of an adequate coronary flow reserve allows 3- to-4-fold increase in the coronary flow to meet the increased metabolic demands. The subendocardial layer is perfused only in the diastole phases and has a lower reserve. Also, it suffers greater systolic compression and oxygen demand due to wall tension, which place it at a higher risk for dysfunction, tissue injury, and necrosis during ischemia [10].

*Cardiac remodelling *is a progressive change in genomic, molecular, cellular, and intercellular structures of cardiac tissue, starting few hours after MI and continuing for years. It finally leads to alterations in size, shape, and function of the heart. Initially, cardiac remodelling is an adaptation of the heart to retain its function after MI. Impaired contractility due to lost myocardium is compensated by the increase in end-diastolic volume in order to restore stroke volume, followed by myocyte hypertrophy, cellular elongation, and proliferation [11]. The global compensative ventricular hypertrophy is not accomplished during 1st year post-MI, therefore, left ventricular (LV) dilatation and thinning continues progressively both in infarct and noninfarct area as a consequence of the volume overload and the increase in global wall tension, according to Laplace's and Frank-Starling law. The increase of wall stress further exacerbates energy imbalance and ischemia, especially in the subendocardial layer, which lead to the additional apoptosis in the affected area. The wall tension, as a result of the dilatation and thinning of ventricular wall, causes even further dilatation. Also, overstretching of the ventricular wall and CMs destroys the functional sarcomeres and further impairs contractility. One of the objectives of myocardial restoration is to stabilize the interaction of sarcomeric CMs and halt or reverse the maladaptive LV dilatation. The timing for cardiac remodelling and the change from an adaptive to a maladaptive process are not clear and may vary. In consequence, the left ventricle undergoes wall thinning, chamber dilatation, and reshaping from the elliptical to spherical form years after onset of MI. Patients develop heart failure [12]. Cardiac remodelling is the key mechanism and predictor of the late adverse outcome after myocardial infarction [13].

## 3. Myocardiac Restoration Methodologies

### 3.1. Cell Therapy

Cell therapy has been applied in clinical myocardial restoration for almost ten years [5]. Though the result is still controversial, some studies have shown the attenuation of the ventricular remodelling. LVEF increased by 2.99%, the infarct size was reduced by 3.5% [14], and cardiac adverse events have been reduced at 2 years followup [15]. It also prevented further dilatation of the left ventricle [16] and improved the systolic function through reduction of the left ventricular end-systolic volume by 4.74% [14].

So far, cardiac cell regeneration has proven to be a relatively safe clinical therapy, and it can be repeatedly performed as an adjunct treatment to percutaneous coronary intervention (PCI) or coronary artery bypass grafting surgery (CABG). There has been no clinical report about tumour formation. Except for myoblasts injection [5], other cell types are not associated with malignant arrhythmias. The increased rate of in-stent restenosis was reported with the intracoronary infusion of the CD133+ fraction of bone-marrow-derived stem cell [17] or granulocyte colony-stimulating factor (G-CSF) [18].

#### 3.1.1. Cell Types in Cardiac Cell Therapy

Many cell types have been investigated (Table 1). The primordial concept in cardiac regeneration was to repopulate the dead CMs with myogenic cells. Until now, skeletal myoblasts (SKMs), CMs, and other progenitor cells capable of differentiation to CMs like embryonic stem cell (ESC), ESC-derived CMs (ESC-CMs), and mesenchymal stem cells (MSCs) have been intensely studied.

*SKMs* showed excellent results in animal [19] and were soon applied in clinical trials [20]. However, poor coupling with host cells and the resulting arrhythmia are major drawbacks of this cell type [5]. Nevertheless, SKMs still attract researchers because of the availability of abundant autologous cells and myogenicity. In an attempt to improve the electrical integration of SKMs with host cells, Connexin 43 gene transfection of myoblasts has reduced the arrhythmogenicity [21].

*The immature CMs* extracted from neonatal rat ventricles have been widely studied in animal models of myocardial infarction. The implantation of CM in post-MI myocardium has shown long survival, coupling, and integration with host CMs, as well as contractile activity [22]. Since the CMs soon terminate hyperplasia and convert to hypertrophy after birth [23], the source of this cell is questionable when considering its therapeutic potential. Methods to retain engrafted cell numbers by an induction of CMs to reenter the cell cycle are being studied [24]. However, the genetically modified cells may pose a safety issue as the risk of tumour formation would arise. Therefore, CMs have not made their way to enter clinical trials so far.

The successful isolation of *cardiac stem cells (CPCs)* from adult myocardium has attracted much interest as a promising cell source. CPCs are capable of proliferating and differentiating to CMs and endothelial cells [25]. CPCs have a high translational potential because they can be harvested by biopsy during open heart surgery and later expanded in vitro before implantation. Intracoronary delivery of cardiac stem cells reduced LV dysfunction after infarction in an animal model [26] and a clinical trial using this cell type has been started. Yet, the contractility and adrenergic receptors have not been found with this cell type and their effect was mainly attributed to paracrine pathway [27].

*Embryonic stem cells* are pluripotent cells which can differentiate to the true cardiac phenotype [28]. Embryonic-stem-cell-derived cardiomyocytes matured and survived upon injection into rat myocardium and are associated with lower risk of tumorigenesis [29]. A recent study by Blin et al. in an immunosuppressed nonhuman primate model showed purified SSEA-1+ ESC-CMs differentiated into ventricular myocytes and reconstituted 20% of the scar tissue, without forming teratomas [30]. Safety of utilization of ESC-CMs remains a limitation as the achieved purity is at the most as 85–90% [31]. Again, ethical issues are still a concern with this cell type.

Recently, *very small embryonic-like stem cells (VSELs)* were found in very small quantity in peripheral blood, umbilical cord blood, and bone marrow (0.02%) [32]. VSELs showed the markers of ESC and cardiac committed phenotype, yet only 7% of purified VSEL cells are able to form spheres that resemble embryoid bodies [32]. They can be expanded and differentiate to the cells of three germ layers similar to ESC. Intramyocardial injection of VSELs in a mouse model of MI, following in vitro expansion and precardiac differentiation, showed differentiation into cardiac phenotype with attenuation of cardiac remodelling and improvement of function [33]. No clinical data with VSELs is available yet.

The introduction of induced *pluripotent stem cells (iPSCs)* using genetic technology may help to mitigate concerns surrounding the ethical and the immunologic issues related to ESCs. iPSCs are stromal cells which are reprogrammed by transfection with a set of pluripotent transcription factors (like OCT3/4. Sox2, c-Myc, Klf-4) that render them pluripotent ECSs [34]. Utilization of these cells for cardiac tissue engineering holds great translational potential, as they could be a patient-specific autologous cell source. iPSCs-derived CMs have shown to have properties similar to ESCs-derived CMs in vitro and, therefore, promise a potential autologous cardiogenic cell source for myocardial regeneration [34]. However, the exact mechanism of reprogramming and their safety relating to transcription factors and transfection vectors are still unclear, a fact that limits their clinical utilization.

**Bone Marrow-Derived Cells (BMCs)**

The early report showed that BMC can transdifferentiate to CMs [35] and it was soon translated for clinical use. However, the subsequent studies showed that the differentiation rate was extraordinarily low [36] and the hematopoietic stem cell fraction followed the hematopoietic fate instead [37]. However, the transplantation of bone-marrow-derived cells, including mononuclear cells, mesenchymal stem cells, and BM-derived circulating progenitor cells, has shown therapeutic effects. Compared to control groups, BMC transplantation improved left ventricular ejection fraction by 3.66%, reduced infarct size by 5.49%, and reduced left ventricular end-systolic volume by 4.80 mL [38].The regenerative potential of bone-marrow-derived stemcells via directcell differentiation cardiac myocytes is not significant [36, 39]. Instead, bone marrow cells implantation improved angiogenesis, antiapoptosis, recruitment of local or circulating stem cells, and secreted bioactive factors to suppress the local immune system and inhibit fibrosis [40].Series of clinical trials have applied bone-marrow-derived stem cells but the results are controversial. So far, intracoronary delivery of bone-marrow-derived mononuclear cells (BMNC) is the most studied [14].

#### 3.1.2. The Fate of Transplanted Cells

Using the methods of intracoronary infusion or intramyocardial injection of suspension of cells, cells are delivered randomly into the myocardium. Therefore, it is impossible to control the distribution of the cells and only 1.3–17.8% of injected cells stay in infarct area [54]. The majority of cells delivered through intracoronary infusion accumulate in border zone, not in the infarct zone (Figure 1) [55].

The retention of cells in the myocardium determines the therapeutic effect. It depends on local responses, including inflammatory changes, upregulation of chemokines receptors and adhesion molecules, and the robustness of the transplanted cells. In fact, cells delivered in a cell suspension will be soon washed out by the venous system, the squeezing of the a heart muscle during systole, and, in case of intramyocardial injection, the leakage through injection holes. Therefore, 50% of injected cells die and only 2–5% are detected after 24 hours [56]. Hence, cell retention becomes crucial in cell therapy [57]. A possible compensation for lost cells would be to deliver a large quantity of cells.

Cell survival is another concern in a cell therapy. Upon transplantation, the majority of cells die in the first 4 days, because of ischemia and inflammation, and only 5–10% of cells survive [58, 59].

#### 3.1.3. The Clinical Outcome of Cell Therapy

The clinical benefit of cell therapy so far is limited and not consistent. In Focus-HF trial, despite having no effect on cardiac function, autologous bone marrow mononuclear cell (ABMMNC) therapy significantly improved quality of life at 6 months, Canadian Cardiovascular Society angina score, and myocardial infusion [48]. Recent results from REPAIR-AMI trial at 2 years followup showed that intracoronary perfusion of autologous bone-marrow-derived progenitor cells (BMCs) reduced occurrence of major cardiac events, including myocardial infarction and revascularization, and improved regional left ventricular contractility of infarcted segments when compared with placebo [15]. On the other hand, the SCAMI trial showed no improvement in function and size of cardiac chamber after MNC intracoronary infusion (381 × 10^6^ cells, 6.1 days after revascularization) when evaluated by cardiac MRI [60], whereas others showed limited improvement of LVEF [14]. The long-term effect is not superior to traditional pharmacological therapies [12, 61] and there is also obvious change in cardiac remodelling [60]. The effect of cell therapy is more related to paracrine effects, which operate to salvage the cells after MI rather than induce new functional tissue in or around the infarct area. Furthermore, the cells are randomly implanted and poorly localized in the infarct area which might make it difficult to build a macroscopic and organized cluster of cells to support scarred myocardium. Therefore, the effect is limited to regional changes, rather than global improvement of cardiac function and structure.

Nevertheless, cell therapy is still an attractive approach because it is feasible in minimally invasively and as a first-line therapy in combination with PCI so far. Cell therapy can be applied in a catheterization laboratory concurrently with primary PCI or weeks later when the acute response subsides, or in combination with CABG.

Many attempts have been made to tackle the disadvantages of cell therapies. Some fractions of bone-marrow-derived cells, including Cd34+ [62], CD45− [52], Cd133 [63, 64], showed better cell survival and retention than nonfractioned bone marrow cells. Overexpressing VGEF [65] or Akt [66] by gene transfection also enhance the cell survival. The treatment of bone-marrow-derived MSCs with platelet-derived growth factor BB (PDGF-BB) resulted in lower rate of apoptosis in vitro and the number of surviving cells has doubled at day 21 posttransplantation [67]. The optimal time for cell injection has been sought for. Because the effect of cell therapy in chronic MI is very limited [56, 68], cells should be transplanted as early as possible after MI to save more cells and prevent remodelling. In contrast, immediate delivery of cells after acute myocardial infarction results in massive cell death because of the intense inflammatory reaction. It was shown that injection of cell between day 5 and 30 after acute MI could offer the better result [38]. In subacute scenario, cells can be delivered during PCI or CABG. Since more than 90% of cells are lost after transplantation, cell therapy requires a large quantity of cells (more than 100 milions with bone marrow cells) to make up for losses and show an improvement of LVEF [14, 69]. Also, the modest improvement of function and geometry of the heart with cell therapy may necessitate the investigation of the other long-term end-points as symptom relief (angina) or major cardiac events rather than cardiac remodelling.

### 3.2. Cardiac Tissue Engineering

The aim of tissue engineering is to replace or support injured tissues through implantation of assembled compounds of cells with degradable biomaterial scaffolds [7].

The matrix provides the physiologic environment, anchors the cells, and protects them from the hostile environment [70]. Optimally, the mechanical support from the biomaterials persists until the new extracellular matrix (ECM) is established. The compound of cells and matrix provides both biological and mechanical support to the ventricular wall, improves the cardiac function, and decelerates the deleterious effect of cardiac remodelling.

The ultimate goal of cardiac tissue engineering is to build a large-scale, artificial myocardium to replace or support the infarcted area. More than a pure three-dimensional structure, the challenging artificial myocardium should be an anisotropic and angiotropic tissue which has rhythm, contractility, and mechanical durability.

#### 3.2.1. The Approaches to Produce Artificial Heart Muscle

With regards to implantation methodology, the artificial constructs can be used as epicardial patch or injectable matrix.

**(1) Cardiac Patch**

**Cell Seeding in Porous Material**

Leor et al. seeded 3 × 10^5^ fetal rat cardiac cells in a 6 × 1 mm 3D preformed alginate scaffold by dropping of cell suspension on top of this dry hydrophilic scaffold. This cell-scaffold construct was cultured in vitro for 4 days and then implanted in rat, subacutely infarcted myocardium by suturing on the heart resulting contracting aggregates. The cells were fed by new network of capillaries which is connected to the neighbouring coronary system of host tissue, which might be induced by growth factors secreted from the implanted embryonic cells.

This degradable porous scaffold degraded after nine weeks and was replaced by the novel ECM. A small number of myofiber bundles was seen embedded among the collagen fibers, but they were not in full integration with the host myocardium. However, the graft attenuated LV dilatation and improved heart function, which might be attributed to the elastic properties of bioartificial grafts and angiogenesis induced by paracrine effects of the embryonic cells and nonspecific immune response against the implanted biograft [71].

**Cell Entrapment**

The disadvantage of cell seeding is the irregular distribution of cells within the porous scaffold. Zimmermann et al. introduced the method to create the construct by mixing cells with the soluble hydrogel of collagen type I and extracellular matrix protein (Matrigel) before its condensation in the casting mold to form a 3D structure [72, 73]. This approach has some advantages over seeding cells in preformed porous materials. Firstly, uniform cell distribution can be achieved. Secondly, it facilitates the mechanical and electrical preconditioning. Zimmerman et al. manipulated further the construct to generate the contractile engineered heart tissue (EHT) by continuing in vitro culture of EHTs for 7 days and then exposing the ring-shape construct to cyclic mechanical strain for another 7 days. Five EHTs were stacked together to increase the thickness before implantation. As a result, it improved the mechanical and electrical integration within host tissue. Neovascularisation with connection of capillaries to the host circulation was also observed. The EHT also contributed to the function of the LV.

**Cell Sheet**

A scaffold-free approach using cell sheets, developed by Shimizu et al. in 2003 [74], was taken by culturing cells in 20–30 nm thick poly(*N*-isopropylacrylamide) dishes. The newly formed cell sheet can be detached from this thermoresponsive cell surface when the temperature is reduced to below 32 degrees Celsius. The three-dimension cell sheet was later created by stacking multiple layers.

The initial limitation of only three layers (80 *μ*m) was later improved by different strategies, including seeding on different types of membrane, stacking in a sandwich fashion with endothelial progenitor cell, or repeated transplantation of triple-layer grafts [75]. The multilayered sheets of cardiomyocytes showed characteristics of cardiac muscle tissue with differentiated sarcomeres and gap junctions as well as macroscopic contractility [76]. Recently, the cell sheet of myoblasts has been applied in clinical trial with encouraging results [43]. However, regardless the ongoing search for safe myogenic cell types, it is still questionable that how many layers of cell can be maximally stacked and if it would be enough to adequately substitute human myocardium.

**(2) Injectable Matrix**

The disadvantages of rigid matrices are the interruption to the continuity of the myocardial architecture, signal transfer, vascularisation, and poor synchronization of the graft's contraction with the host heart [77, 78]. The liquid matrix has some advantages over the rigid materials. It can be produced from synthetic, natural, or decellularized materials. It can comply with the host architecture as a framework to scaffold the large damaged structure in order to prevent remodeling. The liquid matrix offers higher cell viability and retention than the cell suspension. Injectable material alone, without cells, can support the ventricular wall and prevent wall thinning [79]. The clinical potential of this approach lies in the capability to deliver matrix to myocardium minimally invasively via either thoracoscopy or transcatheter. Kofidis et al. showed the better graft/infarct area ratio with injectable matrix + cell (45.5 ± 10.8%) compared with cell alone (29.1 ± 6.7%) in a mouse myocardial infarction model. The fractional shortening in the treated group (27.1 ± 5.4) was also significantly better than in the control group (11.9 ± 2.4) [68]. Singelyn et al. reported injectable matrix from decellularized porcine myocardial tissue which could gel upon injection into rat myocardium and preserve LV volume and ejection fraction [80, 81].

#### 3.2.2. Biological Patch for LV Volume Reduction Surgery

Recently, Miyagi et al. introduced an approach which could be perceived as intersection between tissue engineering and surgical ventricular restoration (SVR) for the treatment of ventricular aneurysm. The study showed that the combination of poly-*ε*-caprolactone-coated gelfoam as a new, reinforced, biodegradable biomaterial, and cytokine/cell treatment (stem cell factor, stromal cell-derived factor-1alpha, bone marrow mesenchymal stem cells) created a viable tissue after SVR and produced better functional outcome than unreinforced gelfoam or modified gelfoam alone [82].

**Whole Heart Reconstruction**

Ott et al. have attempted at rebuilding or reengineering the wholeheart, through decellularization of whole rat hearts and preservation of their three-dimensional structure and vasculature. This acellular heart was reseeded with cardiomyocytes and endothelial cells (ECs) and maintained for up to 28 days by coronary perfusion in a bioreactor. This construct showed macroscopic contraction with very low ejection fraction, similar to 2% of adult or 25% of 16-week fetal heart function [83]. Even though, this approach does not guarantee the reconstruction of the real structure of myocardium. As such, randomly repopulating the decellularized heart with contractile cells might not be sufficient to make it function significantly.

#### 3.2.3. Vascularization of the Grafts

The heart is an angiotropic organ. It relies on the dense network of vessels to meet its very high metabolic demand. There is almost one capillary for every myocyte and the resting blood flow is about 1 mL/gram of heart muscle per minute and it can be increased to three or four times of normal. Therefore, the complex coronary system including the large epicardial coronary arteries, followed by penetrating arteries and capillary plexus, is required to maintain the function of the heart [10].

Any artificial construct with high fidelity to nature needs to incorporate this feature in its structure. Usually, the thickness of construct is less than few hundred microns because the cells in the construct only survive within 100–200 *μ*m from the nearest capillary by the diffusion of oxygen, nutrition from the medium and later host tissue. As the thicker artificial myocardium is required to support the ventricular wall, it is critical to prevascularize the construct before implantation [84].

Many vascularization strategies at various scales have been applied in CTE. Pretreatments of the scaffold with fibroblast [85], triple coculture cells (fibroblast, endothelial cell, and cardiomyocytes) [86], and ascorbic acid [87] have shown better cell survival and promoted vascularization. The composite cell sheet, fabricated by sandwiching endothelial cells between the cell sheets, showed improvement in formation of capillary structure [88].

The abundant blood vessels and availability of omentum was also exploited in order to mature a vasculature in scaffolds [89, 90].

The pedicled grafts would offer clinically relevant vascularization of larger scale engineered tissues as the artificial arterial or venous pedicle can be connected to the host coronaries or aorta to maintain consistent perfusion after implantation. The cellular viability and metabolism could be enhanced by seeding in the pulsatile perfusion culture platform introduced by Kofidis et al. [91]. The chamber was designed with the core vessels of natural origin as rat aorta, embedded in scaffold made of fibrin glue and neonatal cardiomyocytes. After 2 weeks of continuous in vitro pulsatile perfusion, a solid block of 8 mm thick was created. Significant improvement of cell survival was seen in perfused chamber and cells concentrated more in the immediate vicinity of core vessel [91]. Morritt et al. introduced the in vivo rat model of AV loop chamber to prevascularize the graft [84]. Neonatal rat cardiomyocytes in Matrigel were implanted with an arteriovenous blood vessel loop into a 0.5-mL patented tissue-engineering chamber, located subcutaneously in the groin which later form a ~2 mm thick, contractile vascularised scaffold [84]. A vein or synthetic graft is used to form a shunt loop between an artery and a vein. The spontaneously contracting product demonstrated organized assembly, highly vascularization and approached thickness of 2 mm [84].

#### 3.2.4. Clinical Application of Cardiac Tissue Engineering

Preclinical studies have shown the benefit of tissue engineering over cell therapy, with the significant increase of LVEF up to 25%–28% [92, 93]. However, the requirement of a surgical procedure has hindered its clinical application. Chachques et al. implanted a 7 × 5 × 0.6 cm construct epicardially in 10 patients after single-vessel CABG left ventricular end-diastolic volume evolved from 142.4 ± 24.5 mL to 112.9 ± 27.3 mL (matrix, *P* = 0.02) versus 138.9 ± 36.1 mL to 148.7 ± 41 mL (no matrix, *P* = 0.57). The scar area thickness progressed from 6 ± 1.4 mm to 9 ± 1.1 mm (matrix, *P* = 0.005) versus 5 ± 1.5 mm to 6 ± 0.8 mm (no matrix, *P* = 0.09) [94]. Recenly, Sawa reported the recovery of cardiac function after implantation of cell sheet in one patient [43]. Cardiac tissue engineering as stand-alone therapy would require the development of appropriate minimally invasive techniques.

### 3.3. Surgical Ventricular Restoration

In case of dilated left ventricle after MI, the functioning tissue has to work more, as Frank-Starling law demands. There is also more tension on the normal myocardium to compensate the work of infarcted area, as per Laplace's law. As a result, the radius of the left ventricle increases, the thickness decreases, and the heart becomes spherical. The orientation of muscle fibres changes towards more horizontal. Subsequently, 15% shortening of muscle fibres results in only 30% of ejection fraction [9].

Reshaping the heart to an elliptical form would be beneficial. Applying this principle, the Dor's procedure resulted in the increase of LVEF by 12.5% and so as the life expectancy of patients [95]. Recently, Ferrazzi et al. introduced the “horseshoe repair” procedure to preserve left ventricular compliance, which resulted in the increase of LVEF by 20% after 6.9 months followup [96]. The results of these surgical procedures highlight the important role of the structure of the left ventricle in myocardial infarction.

## 4. Conclusion

The cardiac architecture is a crucial target in restoration of cardiac function after myocardial infarction. Cells versus scaffolds alone might not be sufficient to restore the complex structure of infarcted myocardium. Though various cell types and strategies have been investigated, the therapeutic effect of cell therapy in myocardial restoration is still limited. Regardless the disadvantages of cell therapy, such as limited cell survival and retention and the unknown mechanism of action, cells alone actually are not the decisive factor for myocardial restoration as they cannot restore the structure of the LV, the helix of the heart, the vortex mechanism, and structural defects which cause the nonischemic expansion after MI. Tissue engineering has tackled some limitations of cell therapy. However, its goals so far are to develop constructs with a mechanical support to the ventricular chamber in order to reduce LV dilatation and provide an environment for transplanted cells favouring cell survival, proliferation, and differentiation. Yet, tissue engineering has not addressed the real anisotropic and helical structure of the heart and its mechanics.

Myocardial restoration cannot be a monotherapy, but rather polytherapy, as is heart failure therapy today. It should be the comprehensive combination between pharmacological therapy, surgical, and interventional procedures, cell therapy, and tissue engineering on a patient-specific basis.

## Figures and Tables

**Table 1:** Cell sources for Cell therapy.

Cell types	Sources	Advantages	Disadvantages	Delivery	Animal model	Outcome	Clinical application	Mechanism	References
*Skeletal myoblast*	Skeletal muscle	Contractile, highly resistant to ischemia, large volume, autologous	Arrythmia, poor coupling with host tissue	Intramyocardial injection, intracoronary infusion	Rat, sheep	LVEF ↑ EDV ↓ LVSP ↑	intracoronary infusion	Myogenesis Paracrine effect	[41–43]

*Embryonic stem cell-derived CMs*	Embryo	Less tumorigenesis, contractile cells	Ethical issue Purification	Intramyocardial injection	Mouse, Rat, Monkey	LVEF ↑ EDV ↓, Wall thickness ↑	N.A	Myogenesis Paracrine effect	[30, 44]

*Bone marrow mononuclear cells*	Bone marrow	Autologous, large quantity	No myogenesis	Intramyocardial injection or intracoronary infusion	Rat, Canine, Pig	LVEF ↑	LVEF ↑ 0–8.1%	Paracrine effect	[45–48]

*Mesenchymal stem cells*	Bone marrow, cord blood, adipose tissue	Differentiate to CMs Paracrine effects Autologous Large quantity	Low differentiation rate	Intracoronary infusion Intramyocardial injection	Rat, Pig	LVEF ↑, EDV ↓, ESV ↓	LVEF ↑ 12%, intracoronary	Myogenesis Paracrine effect	[49–51]

*VSELs*	Bone marrow	Pluripotent as embryonic stem cell Autologous	Small quantity	Intracoronary delivery, Intramyocardial injection	Mice	LVEF ↑ Wall thickness ↑	N.A	Myogenesis Paracrine effect	[52]

*Cardiac Stem cells*	Heart muscle	Contractile Paracrine effect Autologous	Small quantity Cardiac biopsy or surgical sampling	Intracoronary infusion, intramyocardial injection	Mice, Rat	LVEF ↑; Wall thickness ↑	Yes	Myogenesis Paracrine effect	[26]

*iPSC*	Dermal fibroblast	Pluripotent, can differentiate to CMs Autologous Large quantity	Gene transfer Tumorigenesis	Intramyocardial injection		LVEF ↑ EDV ↓ ESV ↓ Cell differentiate in vitro and in vivo to CMs	N.A	Myogenesis Paracrine effect	[53]

VSELs: very small embryonic like stem cells; iPSC: induced pluripotent stem cells; CMs: cardiomyocytes; LVEF: left ventricular ejection fraction; EDV: end diastolic volume; LVSP: left ventricular systolic pressure.
