# Erratum to “Myocardial Restoration: Is It the Cell or the Architecture or Both?”

**DOI:** 10.1155/2012/678485

**Published:** 2012-06-03

**Authors:** Duc Thang Vu, Eliana C. Martinez, Theo Kofidis

**Affiliations:** ^1^Department of Surgery, Yong Loo Lin School of Medicine, National University of Singapore, Singapore 119228; ^2^Department of Cardiac, Thoracic and Vascular Surgery, National University Health System, 1E Lower Kent Ridge Road, NUHS Tower Block, Level 8, Singapore 119228

This erratum corrects the listing of authors for the above paper published in this journal. The authors' list should also have included “*Dr*. *Eliana C*. *Martinez*, *M.D.*, *Ph.D.*, *from Department of Surgery*, *Yong Loo Lin School of Medicine*, *National University of Singapore*, *Singapore*,” and the authors list should be read in the correct order as previously mentioned.

